# Foreword from FPH President Professor Kevin Fenton and immediate past-President Professor Maggie Rae

**DOI:** 10.1093/pubmed/fdac111

**Published:** 2022-11-21

**Authors:** Kevin Fenton, Maggie Rae

**Affiliations:** Faculty of Public Health; Faculty of Public Health

As the current and immediate past-President of the Faculty of Public Health we have both had the immense honor of serving an organization, and a committed membership, which has driven forward impactful work to protect and improve the public’s health for the past half a century.

For the past 50 years, the Faculty has worked in accordance with our core charitable objects—to promote for the public benefit the advancement of knowledge in the field of public health; to develop public health with a view to maintaining the highest possible standards of professional competence and practice; and to act as an authoritative body for the purposes of consultation and advocacy in matters of educational or public interest concerning public health.

This special edition of our Journal of Public Health—a journal which first entered publication over 100 years ago as the Journal of State Medicine—brings together a collection of papers reflecting these objects and examining the journey that the Faculty, and the public health profession, has been on over the past 50 years.

Much has changed since the first meeting of the Faculty in 1972, not least our name; but whilst the methods and science of public health has constantly evolved to meet contemporary challenges, the commitment of our members to protecting and promoting health for all in society—working to identify and remedy the health inequalities that impact so severely on the most vulnerable—remains the same. In turn, the Faculty, whilst evolving alongside the public health profession, holds the same commitment to supporting our members as they deliver this critical work as it did at it is inception.

Guided by our core principles, the Faculty has worked with our members to achieve an astonishing amount during the past 50 years. Born from a recommendation of the 1968 Royal Commission on Medical Education to create an organization to assume ‘a major role in the training of those who practice the field of community medicine,’ this role has been fulfilled through the Faculty’s training curriculum, examinations and continuing professional development program, and only last year the Faculty received the ASPHER Deans’ and Directors’ Award in recognition of our outstanding training scheme.

Outside of our training function, the Faculty has supported the highest standards of public health practice through overseeing recruitment panels for public health specialists, producing guidance on the ‘gold-standard’ for a public health system and workforce, and expanding our membership to include those from a broad range of multidisciplinary backgrounds.

The Faculty has also sought to promote the best conditions for health to flourish across the UK and overseas though it is policy and advocacy function; advising and holding Government to account on proper arrangements and resourcing for public health systems across the UK, action on health inequalities, and on other specific policy issues including climate change, Brexit and drug policy.

None of this work would have been possible without the support of the Faculty’s membership, and this remains the case as we join together as a profession to address the priorities of the present and the future.

The health inequalities that were coming into sharp focus at the Faculty’s inception in 1972 with landmark work from Hart, Whitehead and Black remain as wicked a problem as they were 50 years ago. We are currently seeing life expectancy stall in the most deprived areas of the UK, rising rates of homelessness, and worsening early years’ health. All of this against a backdrop of a global pandemic, international conflict, and a climate emergency threatening to decimate planetary and human health.

So whilst many papers in this collection look back on the achievements of the Faculty and the public health profession over the past 50 years, it is also right that many of them look to the future and the challenges we face in the coming decades.

We are pleased that the papers in this collection come from professionals working across the entire public health system, with contributors delivering work at local, regional, national and international level across all areas of public health practice; health protection, health improvement, in health services, in academia and elsewhere in civil society.

Several of the papers highlight the importance of partnership in meeting the great challenges of our times. The interconnectedness of modern society makes this cross-system working more critical than ever. Not only must we draw from a multidisciplinary workforce, but we must work with partners in government, industry, media and the voluntary sector to come together to secure the best possible health for future generations.

We thank all authors who have contributed papers to this collection, and a particular thanks to Dr. Farhang Tahzib for his work in drawing together this important reflection on 50 years of the Faculty of Public Health. We also thank Faculty members for your support and dedication over the past 50 years, particularly those who have volunteered their time and expertise to work directly with us; the Faculty would not be in the strong position it is today without your generosity and commitment to improving the public’s health.

The Faculty of Public Health looks forward to the next 50 years of working together to drive forward better health for all.

**Professor Kevin Fenton**

**Professor Maggie Rae**

## Notes

**Kevin Fenton**, Professor

**Maggie Rae**, Professor

